# Genomic analysis of antibiotic resistance and virulence factors in the uropathogen Citrobacter koseri

**DOI:** 10.1099/mgen.0.001751

**Published:** 2026-06-15

**Authors:** Craig Stephens, Braden Christenson, Emily Patacsil, Ananya Velpuri, Andrew Agustin, Alanna Conrad

**Affiliations:** 1 Department of Biology, Santa Clara University, Santa Clara, CA, USA; 2 Department of Public Health, Santa Clara University, Santa Clara, CA, USA; 3 Student Medical Services, Santa Clara University, Santa Clara, CA, USA

**Keywords:** *Citrobacter koseri*, pathogenesis, pathogen genomics, urinary tract infections (UTI)

## Abstract

*Citrobacter koseri* is a significant bacterial cause of community-acquired urinary tract infections in female college students. Given limited previous attention to genomic analysis and molecular epidemiology of this species, whole-genome sequencing was carried out for eight uropathogenic *C. koseri* clinical isolates. Six complete and two draft assemblies are presented. Genome organization was largely conserved between isolates, and comparative analysis with archived genomes suggests that more than 90% of *C. koseri* genes are conserved across the species. These isolates contained relatively few mobile genetic elements (plasmids, prophage, transposons and insertion elements) and extensive antiphage defence systems. Other than a single conserved chromosomal β-lactamase gene, little or no acquired antibiotic resistance was observed. Clues to urovirulence were sought among genes conserved in *C. koseri* but not found in other *Citrobacter* genomes, suggesting roles for iron acquisition, motility and adhesion.

## Data Summary

The *Citrobacter koseri* genome sequencing data, assemblies and annotations described here can be accessed as follows: UTI-18-27 [NCBI accession CP199669, SRA SRX29590936 (Illumina) and SRX29590935 (Nanopore)]; UTI-22-01 [NCBI accession CP199716, SRA SRX29593183 (Illumina) and SRX29593182 (Nanopore)]; UTI-23-21 [NCBI accession CP199717, SRA SRX29593185 (Illumina) and SRX29593184 (Nanopore)]; UTI-23-42 [NCBI accessions CP195891 (chromosome) and CP195890 (plasmid); SRA SRX29606890 (Illumina) and SRX29606889 (Nanopore)]; UTI-23-58 [NCBI accession CP195889, SRA SRX29606892 (Illumina) and SRX29606891 (Nanopore)]; UTI-24-29 [NCBI accession CP196452, SRA SRX29606894 (Illumina) and SRX29606893 (Nanopore)]; UTI-24-68 (NCBI accession JBPUTC000000000, SRA SRX29625701) and UTI-24-81 (NCBI accession JBPUTD000000000, SRA SRX29625702).

**Impact Statement**

In a long-term study of causes of urinary tract infections (UTIs) in female college students, we found the bacterium *Citrobacter koseri* to be the third-most common pathogen. Perhaps because it appears to be a rarer cause of community-acquired UTIs in the general population, this species is poorly understood. Analysis of the eight *C. koseri* genome sequences presented here, including six fully assembled genomes, shows relatively high conservation and low levels of mobile genetic elements, accompanied by limited antibiotic resistance. Several potential virulence factors are present that parallel uropathogenic *Escherichia coli* and are distinct from non-uropathogenic *Citrobacter* species. The comparative genomic analysis presented here sets the stage for further functional analysis of virulence factors in this species.

## Introduction

Urinary tract infections (UTIs) are among the most common bacterial infections among women, who experience the vast majority of uncomplicated community-acquired UTIs [1,2]. This is especially true among women aged 15–29 [1], which includes most college students. Effective prevention and treatment of UTIs may be facilitated by understanding the causal agents of these infections. The majority of community-acquired UTIs are caused by *Escherichia coli*, but other uropathogens are significant in different contexts. Members of the Gram-negative bacterial genus *Citrobacter,* and particularly *Citrobacter koseri* (formerly *Citrobacter diversu*s), are relatively common hospital-acquired uropathogens [3,4]. We have found *C. koseri* to also be a significant cause of community-acquired UTIs in young adult (college-aged) women. To gain insight into the biology and epidemiology of *C. koseri*, we present the genotypic characterization of eight isolates obtained from female college students with clinically confirmed UTIs. These sequences offer potential insights into genome dynamics, antibiotic resistance and urinary tract virulence in this species.

*C. koseri*, a member of the family *Enterobacteriaceae*, is a Gram-negative, motile, facultative anaerobe with relatively simple heterotrophic growth requirements [5]. In addition to its role in UTIs, *C. koseri* has been associated with neonatal and paediatric sepsis, meningitis and other conditions [6,8]. More than 40 complete and 200 draft genome sequences of putative *C. koseri* isolates have appeared in GenBank in the past decade. However, accurate clinical differentiation of the 11 *Citrobacter* species can be error-prone, and many of the deposited *C. koseri* genomes have little or no documentation of the source and clinical context from which isolates were obtained. To date, one publication has presented a complete genome sequence of a *C. koseri* isolate [9], which served as a starting point for comparative genomic analysis of potential virulence factors. We build on that work by presenting here eight new *C. koseri* genomes from clinical isolates of known provenance, six of which are fully assembled. These isolates reveal 4.6–4.8 Mbp genomes with relatively high levels of conservation, few plasmids or other mobile genetic elements and little acquired antibiotic resistance. Such genomic stability distinguishes *C. koseri* from uropathogenic *E. coli*, but *C. koseri* genomes, nevertheless, displayed many similar potential virulence factors to uropathogenic *E. coli* [10]. This analysis sets the stage for further experimental analysis of urovirulence by this underexplored pathogen.

## Methods

### Strains and media

Urine samples from patients (college students) exhibiting UTI symptoms, including a painful or burning feeling while urinating, were obtained over a 6-year period from 2018 to 2024 from Santa Clara University’s on-campus Cowell Student Health Center, after completion of informed consent by the participant. Participants were provided a detailed protocol on collection of a clean-catch urine sample. A sample of the urine specimen was then extracted for in-house use, prior to shipping the main specimen to an off-site clinical diagnostic laboratory. (The eight *C. koseri* isolates described herein were obtained from cultures confirmed by the clinical diagnostic lab to contain *C. koseri*.) Samples were vortexed to resuspend any sediment, and 10 µl of urine was streaked onto CHROMagar Orientation agar [11]. Plates were incubated at 37 °C for 18–30 h. When colonies were present at >10,000 c.f.u. ml^−1^, the dominant colony type(s) was/were re-streaked for isolation. Species identity was confirmed using the API-20E system (Biomerieux), and subsequent genomic DNA sequencing and comparison to the National Center for Biotechnology Information (NCBI) RefSeq database.

### Phenotypic testing

Each *C. koseri* isolate was phenotypically characterized for antimicrobial susceptibility by Kirby–Bauer disc diffusion assays, using guidelines from the manufacturer (Hardy Diagnostics). Antibiotics tested included β-lactams (ampicillin, cephalothin, amoxicillin/clavulanic acid), aminoglycosides (gentamycin, kanamycin, streptomycin), chloramphenicol, quinolones (nalidixic acid, ciprofloxacin, norfloxacin), macrolides (azithromycin), nitrofurans (nitrofurantoin), tetracyclines, sulfonamides (sulfamethoxazole) and trimethoprim. Motility was examined in semi-solid motility agar.

Isolates were tested for flagellar motility by phase contrast microscopy, and on semi-solid motility agar (0.3% agar) [12]. Isolates were also inoculated on the surface of swarm agar (1.5% agar) to test surface-based motility [13].

### DNA sequencing and assembly

Genomic DNA was prepared from broth-grown cultures using the Nucleospin Microbial DNA Mini isolation kit (Macherey-Nagel). DNA preparations were assessed by agarose gel electrophoresis, UV spectroscopy and/or Qubit fluorometry. Library preparation and sequencing with the Illumina MiSeq platform followed manufacturer recommendations. 150 bp paired-end reads were trimmed based on length and quality using BBDuk. Long-read sequencing on the Oxford Nanopore Technology (ONT) MinION instrument followed the native genomic DNA barcoding sequencing protocol (protocol LSK108, ONT). MinION data were processed in MinKNOW (ONT). Combined assembly of long and short reads was done with the Unicycler hybrid assembler (version 0.4.8) [14] on the Galaxy web-based bioinformatics platform [15].

### Computational analysis of genomes

Assembled genomes were analysed using tools from the Center for Genomic Epidemiology (https://cge.cbs.dtu.dk/services/), including ResFinder (ver 4.7.2) for identifying acquired antibiotic resistance genes [16]; PlasmidFinder (ver 2.0.1) to identify potential plasmid replicons [17]; MobileElementFinder (ver 1.0.3) to identify potential transposons (Tns) and other mobile genetic elements [18] and VirulenceFinder to identify potential virulence factors [19,20]. Other genome analysis software used included IslandViewer 4 for identification of genomic islands [21], PHASTEST for prophage identification [22], DefenseFinder for identification of antiphage defences [23] and Ori-Finder 2022 for identifying the origin and terminus of chromosomal replication [24].

**Fig. 1.**
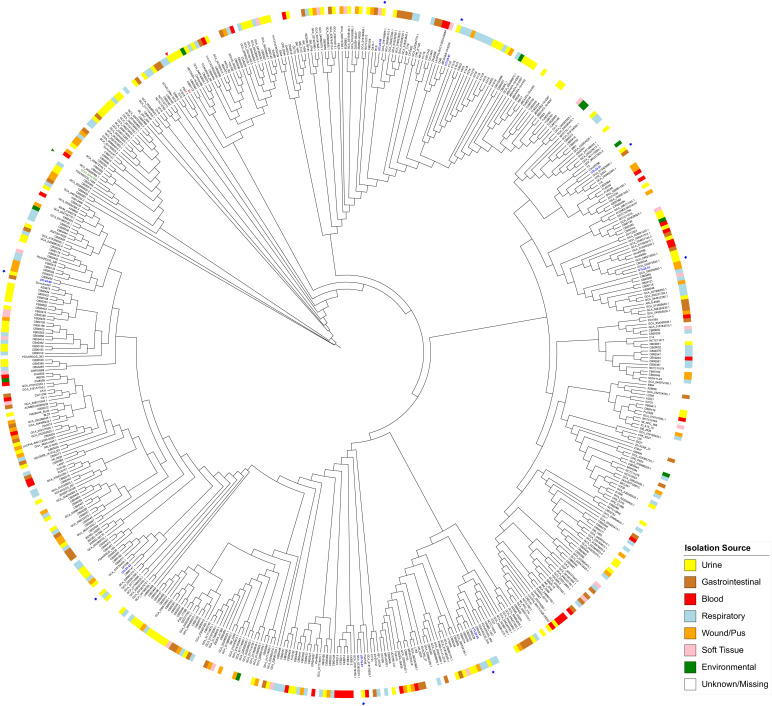
Phylogenetic tree for *C. koseri* based on core genome comparison. 560 assembled *C. koseri* genomes (including the eight described in this work) retrieved from GenBank were annotated using Prokka v1.14.6 [25], and pangenome analysis was performed using Panaroo v1.3.4 [26] with a ‘strict’ cleaning mode and 95% sequence identity threshold for core genes. Phylogenetic reconstruction of the core gene alignment from Panaroo was performed by IQ-TREE v3.0.1 [27]. The best-fit nucleotide substitution model was selected by ModelFinder. A stochastic search algorithm was used to infer maximum likelihood, and nodal support was assessed via 1,000 ultrafast bootstrap replicates. The best-fit model was visualized and annotated using iTOL v6 [28]. Isolate sources are colour-coded, and the SCU isolates are marked by ‘*’. Other genomes noted in the text are indicated by arrows. The tree is unrooted, and branch lengths are ignored for clarity.

To evaluate genomic diversity of *C. koseri*, 606 assembled genomes (including the eight described in this work) were retrieved from GenBank using NCBI Datasets CLI v16.x and annotated using Prokka v1.14.6 [25]. Pangenome analysis was then performed on the annotated assemblies using Panaroo v1.3.4 [26] with a ‘strict’ cleaning mode and 95% sequence identity threshold. The pangenome was characterized as core genes (99%≤strains≤100%), soft core genes (95%≤strains<99%), shell genes (15%≤strains<95%) and cloud genes (0%≤strains<15%). Phylogenetic reconstruction of the core gene alignment from Panaroo was performed by IQ-TREE v3.0.1 [27], and the best-fit nucleotide substitution model was selected by ModelFinder. A stochastic search algorithm was used to infer maximum likelihood, and nodal support was assessed via 1,000 ultrafast bootstrap replicates. The best-fit model (Fig. 1) was visualized and annotated using iTOL v6 [28]. A custom Python v3.x script utilizing a Biopython library was executed to label tree leaves with NCBI assembly accessions or isolate strain names, if applicable. An isolation source dataset was also created from GenBank metadata to group isolates into eight categories: urine (keywords: urine, urinary, UTI, catheter, cysto, clean-catch), blood (keywords: blood, platelets, septicemia, fever patient, culture), gastrointestinal (keywords: stool, rectal, feces, gut, rectum, anal, anus), respiratory (keywords: sputum, BAL, tracheal, lung, broncho, sinus, nasal), wound/pus (keywords: wound, abscess, ulcer, pus, ulcus, pressure), soft tissue (keywords: tissue, skin, heel, back, flank, vagina), environmental (keywords: wastewater, sludge, urban, environmental, grub, food, dairy) and unknown/missing (keywords: N/A, unknown, missing, hospital, patient, clinical, not provided). Genetically redundant isolates were removed to prune the tree to a final 560 unique leaves.

Snippy v4.6.0 [29] was used to map the Santa Clara University (SCU) isolates against the *C. koseri* NJ genome to identify SNPs. Recombination filtering was performed by Gubbins v3.2.1 [30] to mask regions of high sequence diversity from horizontal gene transfer events. IQ-TREE v3.0.1 [27] was used to infer maximum likelihood through a stochastic search algorithm with an applied ascertainment bias correction (+ASC). 1,000 ultrafast bootstrap replicates were used to test branch support. The .contree file output was visualized and annotated in iTOL v6 [28]. Pairwise SNP distances were calculated from the Gubbins-filtered alignment using snp-dists v0.8.2 [30].

The Phylogenetic Profiler tool in the Joint Genome Institute (JGI) Integrated Microbial Genomics (JGI-IMG) platform [31] was used to identify genes that are highly conserved in *C. koseri* genomes and not present in other *Citrobacter* species genomes (‘*C. koseri* unique genes’). The genomes compared were required to be high-quality, complete assemblies as characterized in JGI-IMG. The following genomes were included: *Citrobacter amalonaticus* (FDAARGOS_122, FDAARGOS_1489, FDAARGOS_165, JCM_1661, Y19), *Citrobacter braaki* (FDAARGOS_253, FDAARGOS_290, FDAARGOS_638, MiY-A), *Citrobacter freundii* (B38, B9-C2, CFNIH2, CRCB-101, ERS2650695, FDAARGOS_549, FDAARGOS_61, NCTC 9750, UMH14), *C. koseri* (661 CKOS, AR_0025, ATCC BAA-895, B1B, CB00046, CB00140, CK778, DNF00568, FDAARGOS_164, FDAARGOS_287, FDAARGOS_393, FDAARGOS_526, FDAARGOS_530, FDAARGOS_86, GED7778C, HAMBI_1287, NCTC_10769, NCTC_10770, NCTC_11076, NCTC_10811, NCTC_11075, PSS7778B, NCTC_11074, YDC582), *Citrobacter pasteurii* (FDAARGOS_1424, UMH17), *Citrobacter portucalensis* (30_2, Cf7303, IOMTU157, P10159)*, Citrobacter werkmanii* (BF-6, FDAARGOS_364, FDAARGOS_616, UMH18) and *Citrobacter youngae* (NCTC_13709). The *C. koseri* unique genes were identified as present in all *C. koseri* genomes used for comparison based on a minimum blast e value of <1×10^−5^ and not present in the other *Citrobacter* genomes used by the same e value criteria. For simplicity, genomic locations of specific genes are reported based on the UTI-23-42 chromosome NCBI Prokaryotic Genome Annotation Pipeline (PGAP) annotation.

## Results and discussion

### Uropathogenic *C. koseri* isolation and genome characterization

In a multi-year study (2018–2024) of the bacterial causes of UTIs in a university student population, *C. koseri* was isolated on nine occasions (9/248 culture-positive urine specimens; 3.6%). This was substantially less often than *E. coli* (184/248 culture-positive cases; 74%) and *Staphylococcus saprophyticus* (32/248 culture-positive cases; 13%), but more frequent than any other species, including *Klebsiella pneumoniae* and *Proteus mirabilis. C. koseri* does not show up in the top 10 bacterial causes of community-acquired UTIs in the general population [2,11], but enumeration of the causes of UTIs in young adult women has not been reported previously to our knowledge. Given that this species has also been linked to paediatric UTIs [6], there may be anatomical, physiological or hormonal features of younger women that predispose them to urinary tract colonization by *C. koseri*.

Among the nine SCU *C. koseri* isolates, two were obtained from the same patient in independent visits 10 days apart (UTI-23-58 and UTI-23-61). Subsequent genomic analysis of these two isolates showed them to be essentially identical; these are treated for this work as a single isolate (UTI-23-58). The genomes of all eight *C. koseri* isolates from the campus clinic were sequenced; the six strains isolated between 2018 and early 2024 were sequenced by a combination of short- and long-read methods, with the output integrated into complete assemblies (Table 1). The two more recent isolates (UTI-24-68 and UTI-24-81) were only sequenced with short-read technology (Table 2). Chromosome sizes for the six fully assembled isolates ranged from 4.66 to 4.80 Mbp (mean=4.73 Mbp). Total *C. koseri* genome sizes, including the SCU isolates, were compared with other *Citrobacter* genomes in the IMG database [31]. *C. koseri* genomes (*n*=25, mean=4.83±0.14 Mb) were significantly smaller (t-test, one-tailed, *P*<0.05) than the genomes of *C. amalonaticus* (*n*=31, mean=5.04±0.28 Mb), *C. braaki* (*n*=21, mean=5.12±0.20 Mb), *Citrobacter farmeri* (*n*=12, mean=5.13±0.22 Mb), *C. freundii* (*n*=128, mean=5.24±0.24 Mb), *C. portucalensis* (*n*=9, mean=4.99±0.13 Mb) and * C. werkmanii* (*n*=7, 5.08±0.13 Mb). The size difference with the genomes of *C. youngae* (*n*=6, 4.90±0.18 Mb) was not statistically significant.

**Table 1.** Complete *C. koseri* genome assemblies

	UTI-18-**27**	UTI-22-**01**	UTI-23**-21**	UTI-23**-42**	UTI-23**-58**	UTI-24**-29**
**Isolation date**	4 Dec 2018	22 Nov 2022	10 Mar 2023	26 May 2023	6 Oct 2023	11 Mar 2024
**Illumina reads used**	1.36×10^6^	2.86×10^6^	3.99×10^6^	3.43×10^6^	2.11×10^6^	4.61×10^6^
**ONT reads used (mean length**)	6.5×10^4^ (6,499 bp)	4.8×10^4^ (4,979 bp)	4.8×10^4^ (4,600 bp)	1.6×10^4^ (7,906 bp)	1.7×10^4^ (5,956 bp)	1.1×10^5^ (7,030 bp)
**Chromosome size (Mb)**	4.66	4.80	4.70	4.79	4.73	4.71
**G+C content (%)**	53.7	53.7	53.8	53.8	53.9	53.7
# of coding regions*	4,455	4,697	4,454	4,412	4,360	4,392
# non-coding RNAs*	107	107	106	114	111	115
**Antibiotic resistance genes††**	*blaCKO-1*	*blaCKO-1*	*blaCKO-1*	*blaCKO-1* *fosA7*	*blaCKO-1*	*blaCKO-1*

* Predicted by PGAP.

† Predicted by ResFinder.

**Table 2.** Draft *C. koseri* genome assemblies

	UTI-24-**68**	UTI-24**-81**
**Isolation date**	10/09/24	11/01/24
**Illumina reads used**	2,988,897	2,234,854
**Contigs >1** kb	19	15
**Largest contig (bp)**	1,218,835	1,771,077
**Mean contig length (bp)**	260,403	317,897
**N_50_ of assembly (bp)**	705,172	807,217
**Total length of contigs (Mb)**	95	4.77
**G+C content**	53.7	53.7
# of coding regions*	4,689	4,429
# non-coding RNAs*	88	85
**Antibiotic resistance genes†**	*blaCKO-1* *fosA7*	*blaCKO-1*

* Predicted by PGAP.

† Predicted by ResFinder.

**Fig. 2.**
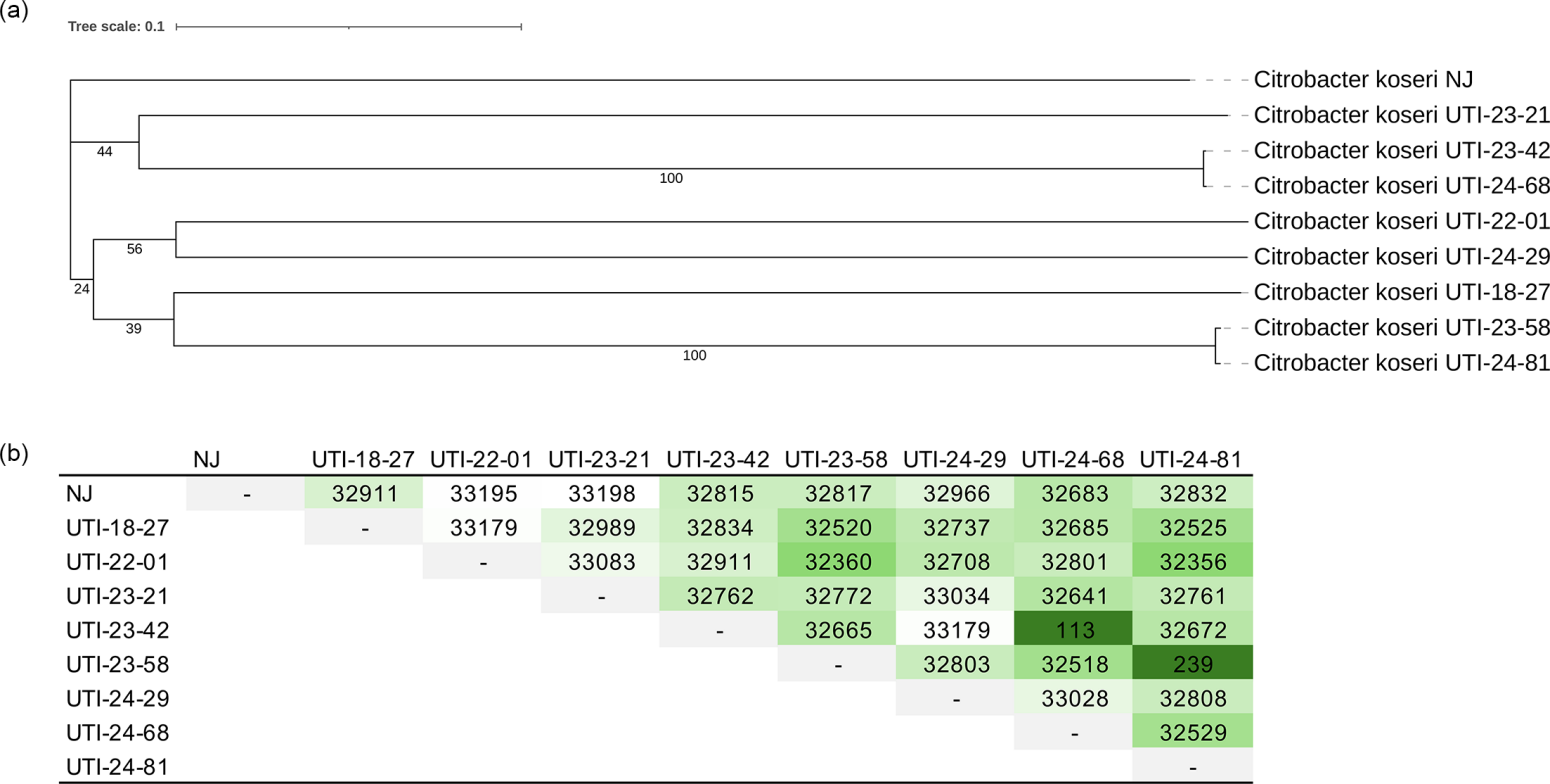
SNP-based phylogenetic distances between SCU isolates. Snippy v4.6.0 [29] was used to compare SCU isolate genomes against the *C. koseri* NJ genome. After recombination filtering using Gubbins v3.2.1 [30], IQ-TREE v3.0.1 [27] was used to infer maximum likelihood through a stochastic search algorithm. 1,000 ultrafast bootstrap replicates were used to test branch support. (**a**) Tree output visualized and annotated in iTOL v6 [28]. (**b**) Pairwise SNP distances calculated using snp-dists v0.8.2 [30]. Darker green colours indicate fewer SNPs.

To assess phylogenomic diversity, the SCU *C. koseri* genome sequences were comprehensively compared to all 560 other sequenced, non-duplicate *C. koseri* genomes available in NCBI at the time of analysis. Fig. 1 shows the SCU isolates (highlighted by asterisks) spanning much of the phylogenetic breadth of the *C. koseri* cluster. To assess more precisely the relationships between the SCU isolate genomes, a phylogenetic tree based on SNPs in the core genome was constructed (Fig. 2). Most of the intrastrain distances were rather extensive, but UTI-23-42 (isolated in 2023) and UTI-24-68 (2024) differed by only 113 SNPs, and UTI-23-58 (2023) and UTI-24-81 (2024) differed by 239 SNPs. The genetic differences between these pairs of SCU isolates, nevertheless, appear to rule out direct transmission chains.

Although *C. koseri* is most commonly known as a urinary tract pathogen (129/560 isolates shown in Fig. 1, 23%), it has been isolated as a commensal or opportunistic pathogen from numerous niches. To test whether urinary tract adaptations might be lineage specific, the origins (if known) of sequenced *C. koseri* isolates were colour coded on the phylogenetic tree in Fig. 1. These included the gastrointestinal tract (57/560, 10%), blood (38/560, 6.8%), respiratory tract (95/560, 17%), wounds (52/560, 9.3%), soft tissue (22/560, 3.9%) and non-human environmental sites (10/560, 1.8%). Consistent with the broad distribution of the eight genomes we analysed, the ability to infect the urinary tract was not limited to a phylogenetically coherent subset of strains.

Panaroo [26] identified a core genome of 3,468 genes that are highly conserved (>99% identity) in all *C. koseri* genomes (~78% of the average *C. koseri* genome). Another 346 (8%) met a more relaxed requirement of 95–99% identity. This conserved core represents a considerably larger fraction (~86%) of the genome than in *E. coli* (45–50%) [32] and *Salmonella enterica* (60–70%) [33].

**Fig. 3.**
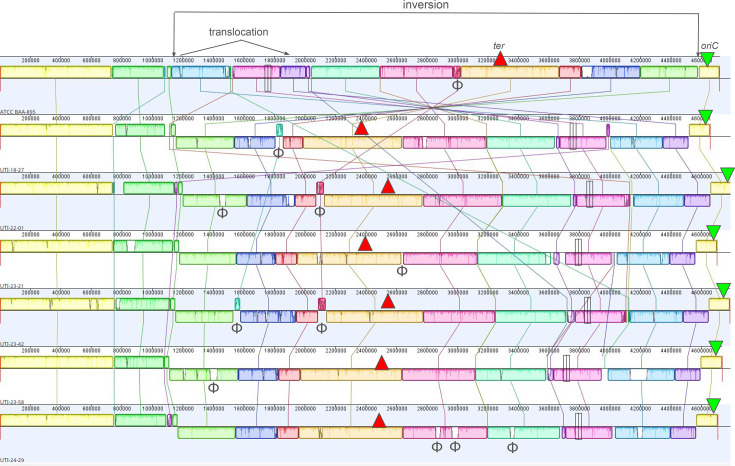
Alignment of *C. koseri* chromosome sequences. Chromosome sequences were aligned with progressiveMauve [75]. For this linear alignment, the numbering of the BAA-895 chromosome was adjusted to start at the same position as the other six, the start codon of *dnaA*. Locally colinear blocks (LCBs) are colour coded. Differences in the *C. koseri* ATCC BAA-895 chromosome structure relative to the other chromosomes, as discussed in the text, are indicated on the top line. Predicted *oriC* sites are indicated with green inverted triangles, and predicted termini of replication are indicated with red triangles [24]. Predicted prophage insertion sites [22] are indicated by Greek letter phi.

Alignments of fully assembled chromosomes from the SCU isolates showed extensive conservation of gene order (Fig. 3), with occasional insertions (ISs) of prophage (Table S1, available in the online Supplementary Material) and genomic islands (Table S2). The predicted origins of replication were roughly 30 kb upstream from the *dnaA* gene. Comparison of these chromosomes to the only previously described fully assembled *C. koseri* genome (ATCC BAA-895, a neonatal meningitis isolate) [9] showed that the ATCC BAA-895 chromosome assembly has two significant rearrangements relative to the six SCU genomes (Fig. 3), possibly due to homologous recombination at rRNA operons. An inversion between the rRNA operons at 1.145 and 4.646 Mb reverses the orientation of the intervening segment in BAA-895 relative to the other six genomes. A translocation also moves 167 kb of DNA between two rRNA operons, originally at one end of the inverted region noted above, and repositions it roughly 700 kb away, again inserted at an rRNA operon and located at 1.837–2.006 Mb in the revised BAA-895 numbering used here. It is unknown whether these apparent differences in the BAA-895 chromosome reflect legitimate genomic rearrangements or assembly artefacts.

Mobile genetic elements, including plasmids, Tn and IS sequences, integrative conjugative elements (ICEs) and prophage can play major roles in the diversification of bacterial genomes [34], and gene acquisition through such elements can shape critical pathogenic traits [35]. Given that *C. koseri* genomes are smaller than other *Citrobacter* genomes, the contribution of mobile elements was examined. Tns and IS elements made minimal contributions to the SCU *C. koseri* genomes (Table 3). A significant exception was the 54 kb ICE containing the colibactin biosynthetic locus (*pks* genes) in UTI-23-42. Colibactin is a polyketide peptide that induces crosslinks in DNA [36]. It acts as a genotoxin and has been associated with colorectal cancer in humans [37]. Colibactin is encoded by the genomes of 15–30% of commensal *E. coli*, primarily phylogroup B2 strains, and is also encoded in some *K. pneumoniae*, *Enterobacter aerogenes* and *C. koseri* isolates [38]. Further examination showed that the *pks* genes were also found in UTI-24-68, albeit fragmented in the latter draft genome among multiple contigs, but were present in less than 2% of all *C. koseri* isolates in NCBI. In *E. coli,* there is evidence that the colibactin biosynthetic pathway may support the production of other polyketide siderophores used for iron acquisition from the host [39].

**Table 3.** Mobile genetic elements and defence systems in SCU *C. koseri* genomes

	UTI-18**-27**	UTI-22**-01**	UTI-23**-21**	UTI-23**-42**	UTI-23**-58**	UTI-24**-29**	UTI-24**-68**	UTI-24**-81**
Plasmids*	0	0	0	1 (9.3 kb, ColRNAI rep)	0	0	2 (34.9 kb, IncX3 rep; 9.3 kb, ColRNAI rep)	0
**Non-plasmid mobile elements†**	8 IS630 (0.1 kb)	1 IS30 (1.2 kb) 11 IS630 (0.1 kb)	8 IS630 (0.1 kb)	1 ICE (54 kb) 1 IS3 (1.6 kb) 1 IS481 (1 kb) 8 IS630-family	8 IS630 (0.1 kb)	5 IS3 (1.6 kb) 1 ISL3 (1.7 kb) 10 IS630 (0.1 kb)	9 IS630 (0.1 kb) 1 IS5 (0.2 kb) 1 IS3 (1.1 kb) 1 ICE (31.8 kb)	3 IS1 (0.4 kb) 2 IS3 (1.6 kb) 8 IS630 (0.1 kb)
**Prophage‡**	one intact (43 kb)	two intact (42 kb, 54 kb)	one partial (27 kb)	two intact (50 kb, 57 kb)	one intact (48 kb)	two intact (48 kb, 60 kb) one partial (36 kb)	two intact (30 kb, 55 kb) one partial (17 kb)	two intact (44 kb, 55 kb)
**Antiphage defence systems§**	Avs CRISPR-Cas3 Gao_TMN Olokun Type II RM	Borvo CRISPR-Cas3 Gabija (3) Gao_Qat Lamassu-Fam PfiAT Shedu ShosTA SoFIC Type I RM Type II RM	BREX CRISPR-Cas3 Gabija Gao_Tmn PD-T7-5 Wadjet	CRISPR-Cas3 DS-24 Lamassu-Fam Menshen PD-T4-2 PD-T7-2 Shedu SoFIC TIR-1 Type IV RM	CRISPR-Cas3 Dpd DS-36 Epona Lamassu-Fam Old exonuclease PD-T4-8 PD-T7-4 Shedu SDIC4 Type I RM	AbiU CRISPR-Cas3 DS-23 PfiAT Retron Shedu Sirona Type I RM Type II RM (2)	CRISPR-Cas3 DS-24 Lamassu-Fam Menshen PD-T4-7 PD-T7-2 Shedu SoFIC TIR-1 Type IV RM	CRISPR-Cas3 PD-Lambda-4 PD-T7-2 PD-T7-5 Shedu SspBCDE TgvAB Type I RM(2) Type II RM
**CRISPR§ repeats**	Total: 45 4.1 : 19 4.2 : 26	Total: 21 4.1 : 14 4.2 : 7	Total: 33 4.1 : 21 4.2 : 12	Total: 21 4.1 : 14 4.2 : 7	Total: 8 4.1 : 2 4.2 : 6	Total: 33 4.1 : 29 4.2 : 4	Total: 20 4.1 : 13 4.2 : 7	Total: 8 4.1 : 2 4.2 : 6

* Predicted by PlasmidFinder [66].

† Predicted by MobileElementFinder [39].

‡ Predicted by PHASTEST [68].

§ Predicted by DefenseFinder [24].

UTI-23-42 and UTI-24-68 both contained a 9.3 kb ColE1-like plasmid found in several other sequenced *C. koseri* isolates (FDAARGOS-287, FDAARGOS-393, and AR-0024) and in numerous *K. pneumoniae* isolates in the NCBI database. This plasmid is likely mobilizable but does not encode antibiotic resistance or potential virulence factors. Sequencing read coverage suggests a copy number of ∼14–20/chromosome equivalent. UTI-24-68 contains a second likely plasmid as well, a 34.9 kb IncX3 replicon designated pUTI-24-68-1 that is >99% identical to *E. coli* plasmid pEC14_35 [40]. Very similar plasmids are found in other *Klebsiella*, *Enterobacter*, *Salmonella* and *Citrobacter* isolates in GenBank. pEC14_35 homologs contain a Type IV secretion system likely used in conjugation but no obvious virulence factors or antibiotic resistance genes. Read coverage suggests a copy number of roughly three per chromosome equivalent.

Prophages are minor contributors to the genomes of the SCU *C. koseri* isolates, with a mean of 78 kb (1.5% of total) and 1–3 prophages present in each (mean 1.6/genome) (Tables 3 and S1). This is unusually sparse; Lopez-Leal *et al*. [41] calculated that genomes of Gram-negative *Proteobacteriales* averaged over three prophages/genome, with the Family *Enterobacteriaceae* even higher, and *E. coli* genomes averaging more than six prophages/genome. Given this, we examined the *C. koseri* genomes for antiphage defences (Table 3) [23]. The only defensive system consistently present in all six genomes was the Type 1F clustered regularly interspaced short palindromic repeats - associated proteins (CRISPR-Cas) system [42,43]. The *C. koseri* CRISPR loci were homologous to the Cas-Y locus structure [44], with two arrays of 28 bp Type 4 repeats (4.1 and 4.2, respectively). Notably, in the closely related UTI-23-58/UTI-24-81 genomes, CRISPR system function is likely compromised by identical frameshift mutations in each of the *cas3f* and *csy2* genes. The number of CRISPR4 repeats in the 4.1 and 4.2 loci varied with each isolate, ranging from a low of two repeats in locus 4.1 in UTI-23-58 and UTI-24-81 to a high of 29 repeats in locus 4.1 for UTI-24-29 (Table S3). Spacer sequences, representing the targets of the processed crRNAs, were generally not conserved between isolates, except for closely related pairs (UTI-23-42/UTI-24-68 and UTI-23-58/UTI-24-81). The Type 1F CRISPR-Cas system is present in most *C. koseri* genomes [137/181 (76%) of isolates in RefSeq] but is rarely found in the genomes of other *Citrobacter* species, suggesting that it was acquired by horizontal transmission following divergence of *C. koseri* from other *Citrobacter*s. blast analysis showed that the *C. koseri* CRISPR locus is most similar to that of *Klebsiella oxytoca* (~75% identity at the nucleotide level), followed by *E. coli* (~73% identity). By restricting acquisition of foreign DNA, the CRISPR system, perhaps supplemented by restriction-modification systems and other defences, may have contributed to the higher genome conservation and smaller genome size of *C. koseri* relative to other *Citrobacter* species.

### Antibiotic resistance

**Fig. 4.**
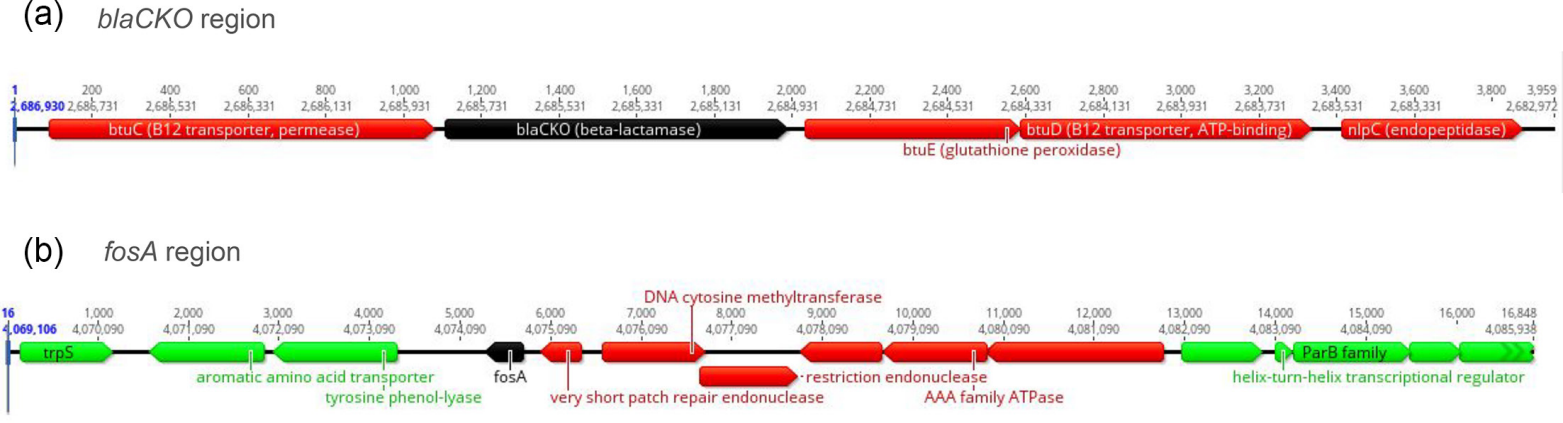
Antibiotic resistance gene loci. (a) *blaCKO* (black arrow) is integrated on the chromosome in the *btu* locus, encoding genes for vitamin B12 uptake. The top numbering is for the DNA segment shown; lower numbering indicates position in the *C. koseri* UTI-23-42 genome. (b) *fosA* (black arrow) is shown in its chromosomal context in UTI-23-42 and UTI-24-68, with numbering shown for the UTI-23-42 chromosome as in (**a**). Green arrows indicate genes conserved in other *C. koseri* isolates, whereas genes coloured black and red are found in a minority of *C. koseri* isolates.

The SCU *C. koseri* isolates were uniformly resistant to ampicillin but sensitive to all other classes of antibiotics tested. Resistance to these β-lactams is due to the chromosomally encoded *blaCKO* gene (also referred to as *blaMAL*), which is universally present in all *C. koseri* genomes examined. *blaCKO* encodes a Class A β-lactamase similar in substrate profile to TEM-1 β-lactamase [45,46]. Clavulanic acid restored sensitivity of the SCU *C. koseri* isolates to the aminopenicillin amoxicillin, consistent with the observation of Petrella *et al*. [46] that BlaCKO is susceptible to inhibition by clavulanic acid. *blaCKO* was overlooked by the analysis of Yuan *et al*. [9], perhaps because it is unique to *C. koseri* rather than a *bla* gene commonly exchanged among *Enterobacteriaceae. blaCKO* is integrated into the *btuCED* locus (vitamin B12 uptake) between *btuC* and *btuE* (Fig. 4a), with no compromise of the surrounding genes. There is no residual evidence of Tn or IS elements on either flank of *blaCKO. blaCKO* may be transcribed as part of the *btuCED* operon; Petrella *et al*. [46] found no evidence of induction of *C. koseri* BlaCKO enzyme activity by β-lactams.

There have been reports of *C. koseri* with extended spectrum β-lactamase (ESBL) genes, including *blaCTX* [47], *blaOXA* [48], *blaKPC* [49,51] and *blaNDM* [52]. Those isolates were not from UTIs, and many were from patients subjected to extended antibiotic treatment for other infections, likely creating intensive selective pressure for acquiring resistance genes.

The UTI-23-42 and UTI-24-68 genomes encoded a novel *fosA* gene most similar (~96% nucleotide identity) to *fosA7* from * S. enterica* [53]. This *fosA* gene was only present in closely related *C. koseri* genomes such as FDAARGOS_287, in contrast to the widespread presence of *fosA* alleles in other *Enterobacteriales*, including *C. freundii* [54]. Fosfomycin resistance was not evident in the two SCU isolates containing *fosA7*, under laboratory conditions using the standard disc-diffusion assay. Wang *et al*. [55] recently reported that *S. enterica* isolates with *fosA7* are also not phenotypically resistant to fosfomycin, urging caution in determining the clinical relevance of this variant. In UTI-23-42 and UTI-24-68, *fosA7* is not part of an identifiable IS element according to MobileElementFinder [18]. *fosA7* represents one end of an 8 kb, seven-gene segment (Fig. 4b) conserved between these two strains and a few other *C. koseri* isolates in the NCBI database. This segment is inserted adjacent to the tryptophanase gene at ∼4.0 Mb on the UTI-23-42 chromosome. FosA encodes a glutathione-S-transferase, and it is conceivable that *fosA7* encodes an enzyme with altered substrate specificity; the expression and significance of *fosA7* and the surrounding region in both *C. koseri* and *S. enterica* warrant further investigation.

No other acquired genes or known mutations leading to antibiotic resistance were observed in the SCU *C. koseri* genomes, consistent with the conclusion of Yuan *et al*. [9] that ‘*C. koseri* had less resistance genes than other species, which may explain why *C. koseri* is considered more susceptible to several antibiotics’.

### Potential virulence factors

Yuan *et al*. [9] suggested numerous virulence factors in the *C. koseri* genome, based on analysis with the VFanalyzer software [56]. The SCU *C. koseri* genomes were scanned with VirulenceFinder to identify potential virulence factors based on homology to known genes with roles in virulence in UTI-causing extraintestinal-pathogenic *E. coli* [19,20] (Table S4). Just five genes were consistently identified as potential virulence factors in these eight *C. koseri* genomes: aerobactin and yersiniabactin synthesis and uptake systems (two genes each), and *nlpI*, encoding a lipoprotein. Aerobactin and yersiniabactin are siderophores used for iron acquisition, an important trait for colonization of the urinary tract by *E. coli* [57,59]. NlpI is an outer membrane lipoprotein involved in cell envelope growth [60]; it is associated with *E. coli* virulence [61], but its precise function is not well understood.

Because *C. koseri* is the only common uropathogen in the *Citrobacter* genus, uropathogenicity may be linked to specific differences in its genome. A comparative genomics approach was used to identify genes consistently present in *C. koseri* genomes but not in genomes of other *Citrobacter* species (see Methods). Just over 100 genes were found that met the inclusion/exclusion criteria (Table 4). The potential relevance of several of these genes to urovirulence is discussed below.

**Table 4.** Genes unique to *C. koseri* genomes

UTI-23-42 locus tag	General function	Gene name and/or function from annotation	Length of product (aa)
ACSR9D_00230	Glycan biosynthesis	β-Galactosamide-α-2,3-sialyltransferase	333
ACSR9D_00385	Unknown	Predicted ATPase	362
ACSR9D_00810	Unknown	Uncharacterized membrane protein YiaA	117
ACSR9D_02375	Metabolism	aroQ: 3-dehydroquinate dehydratase	150
ACSR9D_03625	Gene regulation	DNA-binding transcriptional MerR regulator	141
ACSR9D_03955	Transport	Outer membrane OprD family porin	422
ACSR9D_04560	Transport	l-fucose permease-like major facilitator superfamily (MFS) transporter	436
ACSR9D_05280	Unknown	Putative AbiEii toxin of type IV toxin-antitoxin system	253
ACSR9D_05285	Unknown	RloB-like protein	228
ACSR9D_05350	Carbohydrate uptake	Monosaccharide ATP binding cassette (ABC) transporter - ATP-binding protein (CUT2 family)	500
ACSR9D_05360	Carbohydrate uptake	Monosaccharide ABC transporter - substrate-binding protein (CUT2 family)	354
ACSR9D_05365	Unknown	–	413
ACSR9D_05370	Metabolism	*mtnK* 5'-methylthioribose kinase	416
ACSR9D_05385	Metabolism	Acireductone dioxygenase apoprotein	180
ACSR9D_05385	Metabolism	*mtnC*: acireductone synthase	229
ACSR9D_05390	Metabolism	Methylthioribulose-1-phosphate dehydratase	204
ACSR9D_06295	Unknown	–	196
ACSR9D_06300	Transport	Porin	361
ACSR9D_06305	Gene regulation	*murR*: RpiR family transcriptional regulator	287
ACSR9D_06600	Gene regulation	Regulatory LuxR family protein	193
ACSR9D_06615	Unknown	XapX domain-containing protein	92
ACSR9D_06625	Gene regulation	DNA-binding transcriptional LysR family regulator	300
ACSR9D_06645	Signal transduction	Diguanylate cyclase (GGDEF)-like protein	520
ACSR9D_06650	Metabolism	Pyrroloquinoline quinone (PQQ)-dependent dehydrogenase (s-GDH family)	475
ACSR9D_06655	Transport	Predicted MFS family arabinose efflux permease	534
ACSR9D_08690	Unknown	–	90
ACSR9D_08705	Iron uptake	Carboxylate-amine ligase	374
ACSR9D_08710	Iron uptake	Flavin reductase (DIM6/NTAB) family NADH-FMN oxidoreductase RutF	187
ACSR9D_08720	Iron uptake	Methyltransferase family protein	214
ACSR9D_08725	Iron uptake	iron complex transport system substrate-binding protein (ABC transporter)	337
ACSR9D_08730	Iron uptake	TonB-dependent receptor-like protein	293
ACSR9D_08730	Iron uptake	TonB-dependent receptor-like protein	386
ACSR9D_08860	Iron uptake	Iron complex transport system permease protein	336
ACSR9D_09205	Adhesion	*mrkA*: major type 1 subunit pilin (major subunit)	202
ACSR9D_09215	Adhesion	*mrkC* outer membrane usher protein for mrk system	828
ACSR9D_09220	Adhesion	*mrkD:* type 1 fimbria pilin adhesin	331
ACSR9D_09225	Adhesion	*mrkF*: type 1 fimbria pilin (minor subunit)	216
ACSR9D_09230	Adhesion/signal transduction	*mrkJ:* EAL domain-containing protein (putative c-di-GMP-specific phosphodiesterase class I)	238
ACSR9D_09235	Gene regulation	Regulatory LuxR family protein	194
ACSR9D_09240	Unknown	Similar to flagellar brake protein	237
ACSR9D_09250	Metabolism	*iucD*: lysine N6-hydroxylase	444
ACSR9D_09255	Iron uptake	*iucC*: aerobactin synthase	577
ACSR9D_09260	Iron uptake	*iucB*: acetyl CoA:N6-hydroxylysine acetyl transferase	283
ACSR9D_09265	Iron uptake	*iucA*: N2-citryl-N6-acetyl-N6-hydroxylysine synthase	593
ACSR9D_09270	Transport	Predicted MFS family arabinose efflux permease	403
ACSR9D_09275	Iron uptake	NADPH-dependent ferric siderophore reductase	298
ACSR9D_10685	Gene regulation	DNA-binding transcriptional LysR family regulator	300
ACSR9D_10720	Metabolism	Saccharopine dehydrogenase family	359
ACSR9D_10725	Metabolism	Putative PIG3 family NAD(P)H quinone oxidoreductase	325
ACSR9D_10730	Metabolism	Nucleoside-diphosphate-sugar epimerase	317
ACSR9D_10775	Iron uptake	ybtE: AMP-binding enzyme	66
ACSR9D_10290	Iron uptake	ybtT: thioesterase component of yersiniabactin synthetase	267
ACSR9D_10790	Iron uptake	irp1: yersiniabactin nonribosomal peptide/polyketide synthase	3124
ACSR9D_11550	Gene regulation	Regulatory LuxR family protein	153
ACSR9D_11555	Unknown	–	147
ACSR9D_12070	Metabolism	l-Lactate dehydrogenase	314
ACSR9D_12640	Unknown	α/β-hydrolase	312
ACSR9D_13280	Unknown	–	43
ACSR9D_13970	Transport	NitT/TauT family ABC transporter, substrate-binding protein	335
ACSR9D_13980	Transport	NitT/TauT family ABC transporter, permease protein	288
ACSR9D_13985	Cell envelope metabolism	Peptidoglycan/LPS O-acetylase OafA/YrhL	379
ACSR9D_14190	Unknown	Stealth-like protein	335
ACSR9D_15180	Iron uptake	Iron complex transport system substrate-binding protein	328
ACSR9D_15185	Iron uptake	Iron complex outer membrane receptor protein	722
ACSR9D_17225	Unknown	Plasmid segregation centromere-binding protein ParR	180
ACSR9D_17255	Motility	*fliL-2*	156
ACSR9D_17260	Motility	*fliK-2*: flagellar hook-length control	357
ACSR9D_17265	Motility	*fliA*	101
ACSR9D_17275	Motility	*fliD-2*: flagellar hook-associated protein 2	442
ACSR9D_17290	Unknown	Hypothetical protein with winged helix-turn-helix domain	218
ACSR9D_17295	Unknown	–	156
ACSR9D_17300	Unknown	–	176
ACSR9D_17305	Motility	*flgL*: flagellar hook-associated protein	305
ACSR9D_17365	Motility	FlgM family anti-sigma-28 factor	90
ACSR9D_17370	Motility	*flgN*: flagella synthesis protein	145
ACSR9D_17385	Unknown	Glycosyl transferase family 9 (putative heptosyltransferase)	295
ACSR9D_17390	Unknown	Lysine-N-methylase	313
ACSR9D_17440	Motility	Flagellar motor switch protein FliM	294
ACSR9D_17470	Unknown	LprI-family protein (lysozyme inhibitor)	329
ACSR9D_17485	Unknown	–	114
ACSR9D_17490	Unknown	–	176
ACSR9D_17500	Unknown	–	63
ACSR9D_17505	Gene regulation	AraC-like DNA-binding protein	251
ACSR9D_17510	Unknown		126
ACSR9D_17515	Adhesion	Major type one subunit fimbrin (pilin)	192
ACSR9D_17510	Adhesion	Fimbrial protein	363
ACSR9D_17535	Gene regulation	DNA-binding NarL/FixJ family response regulator	231
ACSR9D_17540	Signal transduction	EAL domain-containing protein	67
ACSR9D_17540	Signal transduction	EAL domain-containing protein	169
ACSR9D_17545	Gene regulation	Regulatory LuxR family protein	195
ACSR9D_17560	Unknown	Histidine ammonia-lyase-family protein	514
ACSR9D_17565	Osmoregulation	Osmoprotectant ABC transport system, permease protein	207
ACSR9D_17570	Osmoregulation	Osmoprotectant ABC transport system permease protein	210
ACSR9D_17580	Metabolism	Ornithine cyclodeaminase	334
ACSR9D_17630	Unknown	–	327
ACSR9D_17635	Metabolism	Methionine synthase (B12-independent)	343
ACSR9D_18860	Transport	2-Hydroxycarboxylate transporter family protein	301
ACSR9D_18865	Unknown	PAS domain-containing protein/ATP binding protein	270
ACSR9D_18870	Gene regulation	Two-component system response regulator CitB	230
ACSR9D_21810	Unknown	–	196
ACSR9D_21825	Unknown	–	235

**Fig. 5.**
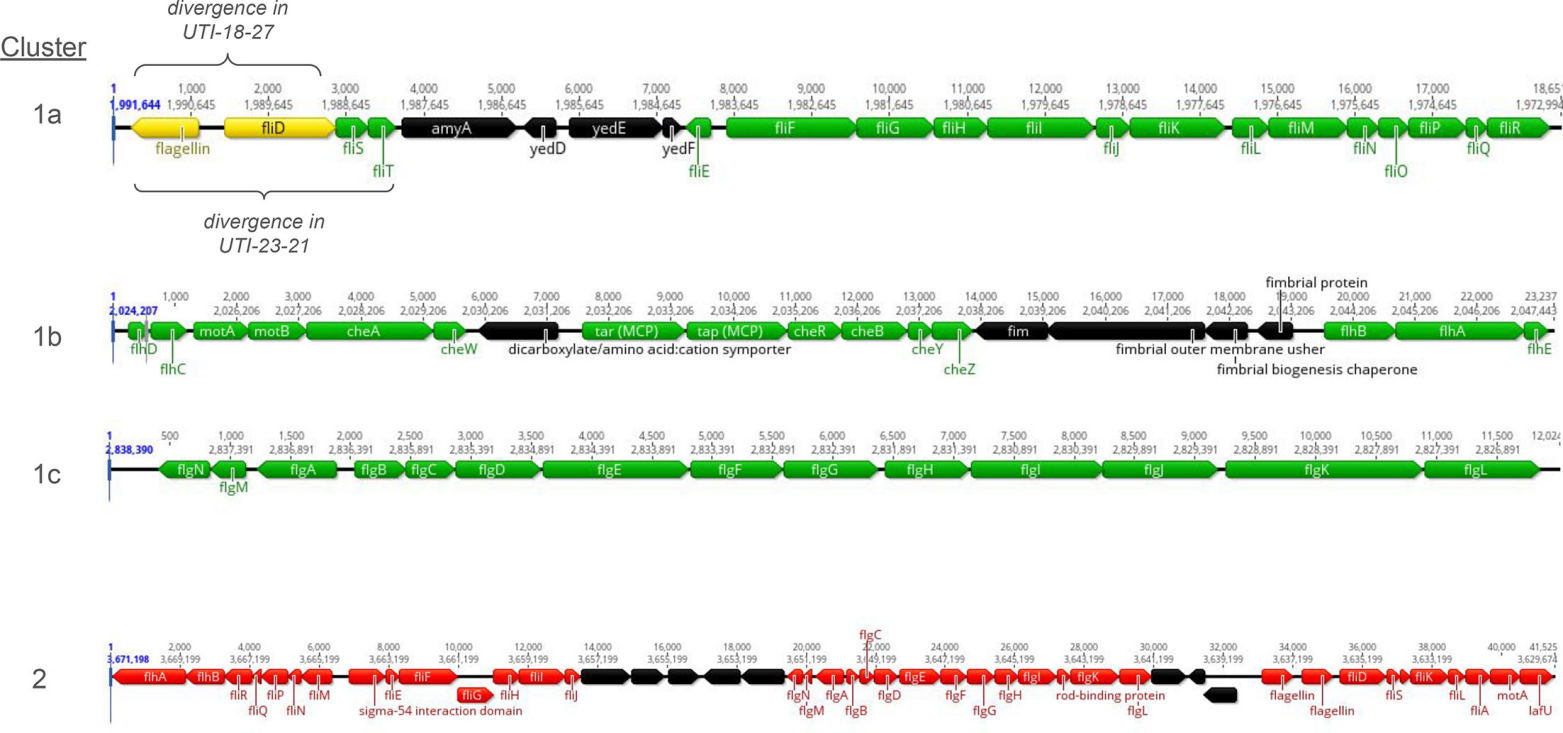
Flagellar gene clusters. For each region, the top numbering is for the DNA segment shown; lower numbering indicates position in the *C. koseri* UTI-23-42 genome. Cluster numbering (shown to the left) is as described in the text. Gene designations are based on annotation, largely based on homology to known flagellar genes in *E. coli* and *S. enterica*. Black genes are not involved in motility functions, based on their annotation. As indicated by the brackets above and below cluster 1 a, the *fliC-fliD* genes are substantially divergent in sequence in the UTI-18-27 and UTI-23-21 genomes relative to the other SCU *C. koseri* isolates; the *fliS* and *fliT* genes in UTI-23-21 are also distinct (see text).

Flagellar motility is relevant to virulence in many bacterial pathogens, including uropathogenic *E. coli*, where flagellar motility facilitates ascent of the urethra to the bladder [62] and penetration of the mucosal lining to contact epithelial cells [63]. Inspection of the *C. koseri* genome revealed that over half of the genes associated with building and operating a flagellum are present in two copies. A complete set of *E. coli/Salmonella*-type flagellar/motility/chemotaxis genes is found in three clusters: Clusters 1a and 1b are in the proximity of 2.0 Mb on the *C. koseri* genome map, with the remainder in cluster 1c at roughly 2.8 Mb (Fig. 5a–c). The genes in these three clusters are 75–85% identical in DNA sequence to homologs in other *Citrobacter* species, suggesting that they are descended from the ancestral *Citrobacter* flagellar system. The mutation reported by Townsend *et al*. [64] to cause a non-motile phenotype in *C. koseri* occurred in the cluster 1a *fliP* gene, implicating the ancestral *Citrobacter* system in swimming motility under laboratory conditions. Seven *C. koseri*-unique genes highlighted in Table 3 are associated with a second genetic region (cluster 2, found at roughly 3.7 Mb on the genome map; Fig. 5d). The cluster 2 genes are only ~50–60% identical at the DNA level to their paralogs in cluster 1 but are >70% identical in DNA sequence to flagellar genes from *Kluyvera* and *Klebsiella* genomes (sister genera to *Citrobacter* within the *Enterobacteriaceae*), suggesting they were derived by horizontal acquisition. Cluster 2 also shows substantial differences in organization relative to cluster 1. The flagellar master transcriptional regulators encoded by *flhCD*, as well as the chemotaxis machinery (*cheABRVWYZ*) and most (11/12) of the sensory methyl-accepting chemotaxis proteins are only encoded in cluster 1.

**Fig. 6.**
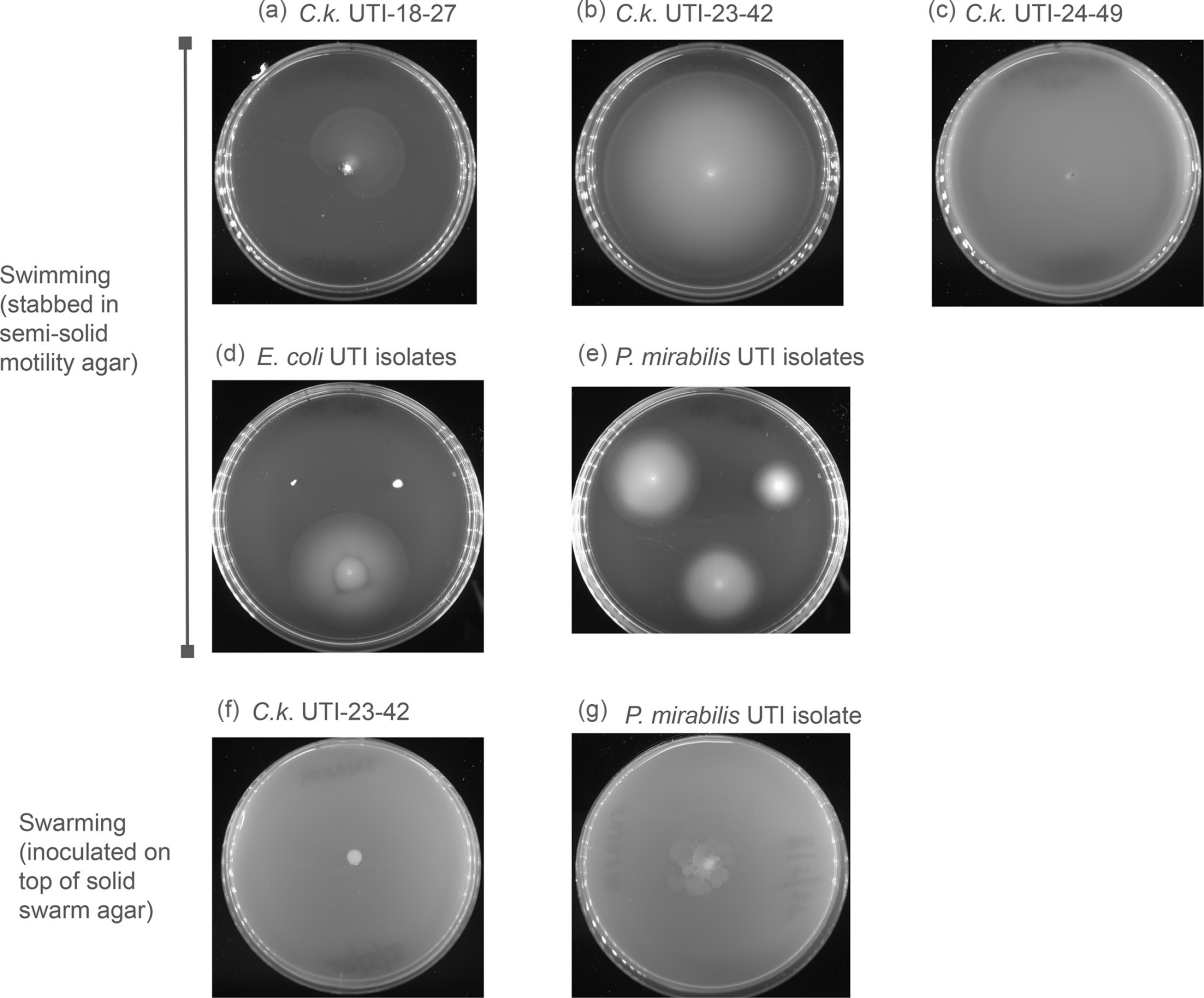
Motility of *C. koseri* isolates. Plates (**a**)–(**e**) are semi-solid motility agar (0.3% agar), photographed 24 h after stabbing in the centre of the plate. Plates (**f**) and (**g**) are swarm agar (1.5% agar) photographed 18 h after inoculation on the surface of the plate. Species and strain designations (where appropriate) are indicated.

When the SCU *C. koseri* isolates were examined for motility by stabbing in semi-solid agar (Fig. 6), all but UTI-18-27 were active, moving through 0.3% agar media more rapidly than uropathogenic *E. coli* isolates. Isolate UTI-18-27 was not motile by microscopic observation in liquid culture, but in semi-solid motility agar, flares of motile cells from the UTI-18-27 inoculation site were common, suggesting that the non-motile phenotype is not genetically stable and that motility can be regained by reversion or further mutation. The genomic basis for the lack of motility in UTI-18-27 is unknown. The *fliC* (flagellin) and *fliD* (filament cap) genes at the left edge of cluster 1a in UTI-18-27 diverge significantly from the other isolates: *fliC* showed only 75% identity and *fliD* only 77% to the homologs in most of the other SCU *C. koseri* isolates, which typically are >99% identical between cluster 1 flagellar gene homologs. Interestingly, UTI-23-21 (a highly motile isolate) also showed replacement of the *fliC-fliD* genes in cluster 1a, with only 64 and 56% identity, respectively, to most SCU *C. koseri* homologs, and even less similarity to UTI-18-27. It is possible that the cluster 1b *fliC-fliD* region is a hotspot for recombination with acquired DNA from related species, potentially affecting both flagellar function and antigenic diversity.

The role of flagellar motility in *C. koseri* uropathogenesis has not been tested, although Townsend *et al*. [64] found that the non-motile *fliP* mutant was hypervirulent in a rat model of *C. koseri* meningitis. Dual flagellar systems have been identified in other pathogenic bacteria, including *Aeromonas hydrophila* and *Vibrio parahaemolyticus* [65,66], in which one flagellar system provides for swimming motility while the other system facilitates ‘swarming’ motility on surfaces and/or under higher viscosity conditions [67]. However, when *C. koseri* isolates were inoculated on the surface of agar media that support swarming of *P. mirabilis* and *C. freundii*, no surface movement was observed.

Sedimentation was observed when *C. koseri* UTI isolates were grown under static conditions, particularly in synthetic urine media; microscopic observation of large aggregates suggests potential for adherence and biofilm formation. Aggregation, adherence and biofilm formation are often mediated by fimbriae/pili. In uropathogenic *E. coli,* Type 1 pili mediate attachment to uroepithelial cells via the mannose-binding function of the FimH adhesin [68]. In uropathogenic *K. pneumoniae*, both Type 1 and Type 3 pili are important colonization factors for the urinary tract [69]. Ong *et al*. [70] have suggested that the *mrkABCDE* operon responsible for Type 3 pilus assembly has a similar role in *C. koseri*. The *C. koseri* genome is rich in annotated fimbrial systems, with at least 14 such gene clusters, but only two fimbrial gene clusters are unique to *C. koseri*. One is the Mrk locus, located at 1.95 Mb in the UTI-23-42 genome, adjacent to the locus for production and utilization of the siderophore aerobactin (see below), another potential virulence trait. The second locus, at 3.7 Mb, is annotated as a Type 1 system, although it is structurally similar to the Mrk locus.

The ability to transition between motility and adhesion is a critical regulatory juncture for many bacterial pathogens and is often mediated by the second messenger cyclic-di-GMP [71]. Multiple putative diguanylate cyclase (GGDEF-like) proteins are encoded by *C. koseri* genomes, including seven in UTI-23-42. The *C. koseri*-unique gene set includes one of these (ACSR9D_06645). Responses to cyclic-di-GMP are typically mediated by EAL-domain proteins, which are also abundant in *C. koseri* genomes; UTI-23-42 contains at least nine. The *C. koseri*-unique genes include *mrkJ* (ACSR9D_09230), an EAL-domain protein associated with regulation of the Type 3 Mrk pili noted above. A second *C. koseri*-specific fimbrial locus also includes an adjacent EAL-domain protein (ACSR9D_17540). Another potentially virulence-relevant EAL-domain protein, albeit not unique to *C. koseri* genomes, is *ycgR* (ACSR9D_10580), which controls flagellar speed in response to c-di-GMP levels [72].

As noted above, the *C. koseri*-specific genes in Table 4 include four multi-gene clusters associated with iron uptake, including synthesis, uptake and processing of the hydroxamate siderophore aerobactin and the polyketide siderophore yersiniabactin. * C. koseri* genomes also contain inner membrane iron uptake systems for ferric citrate (*fec* genes) and ferric hydroxamate complexes (*fhu* genes), the latter of which is needed for aerobactin utilization. The aerobactin/Fhu and ferric citrate iron acquisition systems have been associated with uropathogenesis in *E. coli* [58]. Yersiniabactin is implicated in virulence in *Yersinia pestis* and * K. pneumoniae* [73] and is necessary for virulence in *C. koseri* TBCP-5362 [9]. *C. koseri* genomes also contain a 20-kb gene cluster encoding the synthesis of the catecholate siderophore enterobactin (*entABCDEFS*) and its uptake and processing to release ferric ions (*fepABCDEG*).

Beyond iron acquisition, it is not evident how the *C. koseri*-unique genes in Table 4 might metabolically adapt the organism for growth in the bladder. *C. koseri* genomes encode genes necessary for transport and metabolism of urea, citrate and d-serine, major organic nutrients available in urine [74], but these pathways are shared by most other *Citrobacter* species as well and thus do not appear to be specialized adaptations for urinary pathogenesis.

Comparative genomic analysis can guide future experimental and epidemiological investigation of *C. koseri* as a urinary tract pathogen, as well as in other invasive contexts. The current study has clear limitations, in that it is based on relatively few, albeit diverse, *C. koseri* isolates derived from a specific type of patient (college-aged women), in a specific geographic region (northern California, USA), and isolated from a specific pathogenic context (uncomplicated cystitis). This study suggests that, relative to *E. coli*, *C. koseri* genomes are more conserved and less likely to acquire antibiotic resistance genes, plasmids, prophage and other mobile genetic elements. Defensive systems such as CRISPR-Cas may be associated with this resistance to incoming genetic material. This work also suggests that, despite this resistance, *C. koseri* genomes display genes differentiating them from other *Citrobacter* species, some of which may be associated with urinary tract pathogenicity, including genes involved in motility, adhesion, intracellular signalling and iron uptake. Further work is necessary to test the generality of these conclusions.

## Supplementary material

10.1099/mgen.0.001751 Table S1.

10.1099/mgen.0.001751 Table S2.

10.1099/mgen.0.001751 Table S3.

10.1099/mgen.0.001751 Table S4.

## Abbreviations

ASC: ascertainment bias correction
Cas: CRISPR-associated protein
CRISPR: clustered regularly interspaced short palindromic repeats
ESBL: extended spectrum β-lactamase
ICE: integrative conjugative element
IS: insertion
JGI-IMG: JGI integrated microbial genomics
NCBI: National Center for Biotechnology Information
ONT: Oxford Nanopore Technologies
PGAP: Prokaryotic Genome Annotation Pipeline
SCU: Santa Clara University
Tn: transposon
UTI: urinary tract infection
